# Myelin injury induces axonal transport impairment but not AD-like pathology in the hippocampus of cuprizone-fed mice

**DOI:** 10.18632/oncotarget.8981

**Published:** 2016-04-25

**Authors:** Junjun Sun, Hong Zhou, Feng Bai, Qingguo Ren, Zhijun Zhang

**Affiliations:** ^1^ Department of Neuropsychiatry, Affiliated ZhongDa Hospital, School of Medicine, Southeast University, Nanjing, China

**Keywords:** demyelination, axonal transport, tau, beta amyloid, remyelination, Gerotarget

## Abstract

Both multiple sclerosis (MS) and Alzheimer's disease (AD) are progressive neurological disorders with myelin injury and memory impairment. However, whether myelin impairment could cause AD-like neurological pathology remains unclear. To explore neurological pathology following myelin injury, we assessed cognitive function, the expression of myelin proteins, axonal transport-associated proteins, axonal structural proteins, synapse-associated proteins, tau and beta amyloid and the status of neurons, using the cuprizone mouse model of demyelination. We found the mild impairment of learning ability in cuprizone-fed mice and the decreased expression of myelin basic protein (MBP) in the hippocampus. And anti-LINGO-1 improved learning ability and partly restored MBP level. Furthermore, we also found kinesin light chain (KLC), neurofilament light chain (NFL) and neurofilament heavy chain (NF200) were declined in demyelinated hippocampus, which could be partly improved by treatment with anti-LINGO-1. However, we did not observe the increased expression of beta amyloid, hyperphosphorylation of tau and loss of neurons in demyelinated hippocampus. Our results suggest that demyelination might lead to the impairment of neuronal transport, but not cause increased level of hyperphosphorylated tau and beta amyloid. Our research demonstrates remyelination might be an effective pathway to recover the function of neuronal axons and cognition in MS.

## INTRODUCTION

Multiple sclerosis (MS) is a progressive neurological disorder of young adults, characterized by myelin destruction and neurodegeneration in the central nervous system (CNS) [1]. Similar to Alzheimer's disease (AD), memory impairment is a common symptom of MS [2, 3]. Previous imaging studies have demonstrated that altered hippocampal measures and decreased hippocampal volume correlate with memory impairment in MS patients [4–6]. And similar results are also found in AD patients [7–9]. Postmortem studies have shown that 53 to 79% of MS hippocampi are detected demyelination and demyelinated hippocampi consist with significant decreases in neuronal proteins essential for the function of neurons [10–12].

Myelin is an important structure in the CNS, which contributes to the fast and effective brain function [13, 14]. Demyelination may cause the change of neuronal proteins and dysfunction of neurons, and lead to cognitive impairment [15]. MS is one of common demyelinated diseases in the CNS. Besides MS, myelin impairment is also detected in the brain of AD patients [16]. Previous image research has demonstrated that the white matter is injured in the brain of AD patients [8, 17, 18]. And the autoantibodies (antibodies against myelin associated proteins) are observed in significant higher titers in AD patients, compared with healthy controls [19]. Furthermore, postmortem studies have shown that demyelination is coexisted with amyloid plaques in the brain of AD cases [20, 21]. And in the AD transgene mice, myelin impairment precedes the appearance of beta amyloid and hyperphosphorylation of tau [22]. However, the relationship between demyelination and AD-like pathology is still unclear.

The cuprizone model is one of the most common experimental demyelination animal models of MS, which is induced through a non-inflammation way [23]. The cuprizone is a drug special to impair the mature oligodendrocyte leading to demyelination, but it does not influence the viability of the neuroblastoma cell line SH-SY5Y cells, microglia, and astrocytes, and the proliferation and survival of oligodendrocyte precursor cells (OPC) [24]. Previous research has showed that cuprizone could induce significant demyelination in the corpus callosum, hippocampus, and so on [25, 26]. Therefore, in this research, we used cuprizone-fed mice as the demyelination model to detect whether demyelination could induce AD-like pathology.

LINGO-1 is a transmembrane protein, which is specifically expressed in oligodendrocytes and neurons [27]. Numerous studies, both *in vitro* and *in vivo*, have showed that antagonist the function of LINGO-1 can promote myelin formation of oligodendrocyte [9, 28–30]. LINGO-1 antibody can promote remyelination and functional recovery in experimental autoimmune encephalomyelitis (EAE) mice [29, 31]. However, LINGO-1 deficiency has no effect on inflammation [29]. All these research has demonstrated that LINGO-1 antagonist is one of the important ways to promote remyelination in the CNS.

In the research, we found that the cuprizone model had mild spatial learning impairment with significant demyelination in the hippocampus. And anti-LINGO-1 had slightly improved the ability of learning and partly increased the expression of myelin basic protein (MBP) in the hippocampus. We also found kinesin light chain (KLC), neurofilament light chain (NFL) and neurofilament heavy chain (NF200) were declined in the cuprizone model, and anti-LINGO-1 treatment could partly improve the expression of KLC, NFL and NF200. Furthermore, the synaptic protein spinophilin was decreased in the hippocampal cortex and it was slightly increased after anti-LINGO-1 treatment. However, we did not find the increased level of beta amyloid, abnormal phosphorylation of tau and neuronal loss, which are important hallmarks of AD. Our research suggests that demyelination may lead to the impairment of neuronal transport, and decreased expression of neurofilament proteins and synaptic protein, but not cause AD-like hyperphosphorylated tau, increased level of beta amyloid and neuronal loss. Furthermore, remyelination may be an effective pathway to recover the function of neuronal axons and cognition.

## RESULTS

### The behaviors of cuprizone-fed mice

The mice were experienced cuprizone administration for about ten weeks, and the behaviors were tested at weeks 9 to 9.5, including the elevated plus maze (EPM), open field test, sucrose preference test and Morris water maze test (MWT). LINGO-1 antibody treatment was began in the third week and continued to the end of behavioral tests without stopping cuprizone administration.

**Figure 1:**
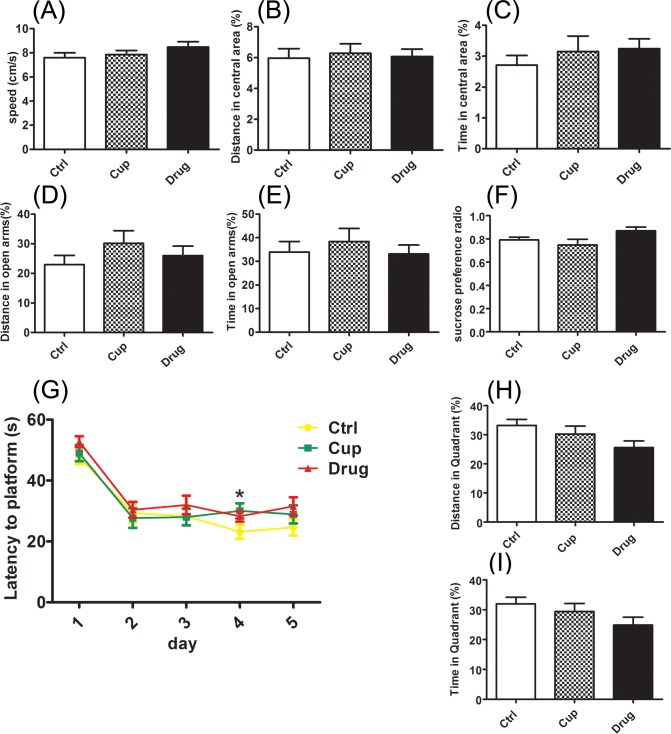
The behaviors of cuprizone-fed mice The behaviors were tested at week 9-9.5, including the open field test **A.**-**C.**, elevated plus maze **D.** and **E.**, sucrose preference test **F.** and Morris water maze test **G.**-**I.** *Denotes statistical significance compared with controls (*P* < 0.05).

The results showed that the cuprizone-fed mice had no anxiety and depression-like behaviors, displayed as that there was no difference between the cuprizone-fed mice and control mice in the open field test (Figure 1A-1C), elevated plus maze (EPM) (Figure 1D-1E), and sucrose preference test (Figure 1F). In MWT, during the training days, cuprizone-fed mice performed worse than the controls, showing as the latency to reach the platform in cuprizone-fed mice were longer than that in the control mice on the fourth day (Figure 1G). And after the LINGO-1 antibody treatment, the latency in cuprizone-fed mice was similar to that in control mice (Figure 1G). On the detecting day, neither the distance nor the time in the platform quadrant was different between the cuprizone-fed mice and control mice (Figure 1H and 1I).

### Demyelination in the hippocampus

**Figure 2:**
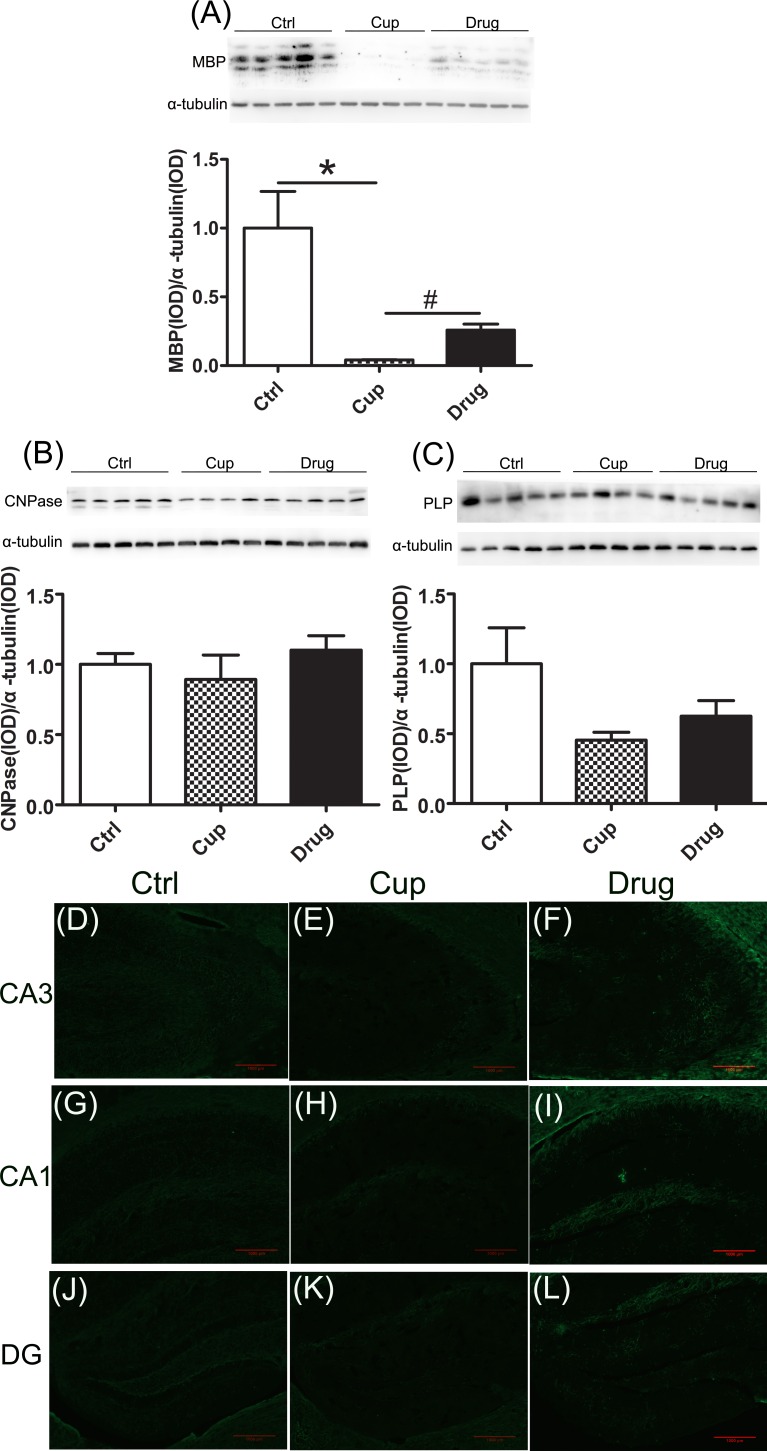
Demyelination in the hippocampus Myelin associated proteins in the hippocampus, including MBP **A.**, CNPase **B.** and PLP **C.**, were detected among the three groups using Western-blot. Figures **D.**-**L.** were displayed the immunofluorescence staining of MBP in subregions of the hippocampus, including Ca3 (D-F), Ca1 (G-I) and DG (J-L). *denotes statistical significance compared with controls (*P* < 0.05). #denotes statistical significance compared with the cuprizine-fed mice without treatment (*P* < 0.05). Images were captured from stained frozen sections using a fluorescence microscope equipped with 10×objectives. Scale bar, 1000μm.

In the hippocampal cortex, we detected the myelin-associated proteins, including MBP, 2′,3′-cyclic nucleotide 3′-phosphodiesterase (CNPase), and proteolipid protein (PLP). We found that the expression of MBP was significantly declined in the cuprizine-fed mice compared with the control group and it was increased in the LINGO-1 antibody treated group (Figure 2A). However, other myelin-associated proteins, including CNPase and PLP, were reduced but not significantly in the cuprizine-fed mice (Figure 2B and 2C). In immunofluorescence staining, we also found decreased expression of MBP in subregions of the hippocampal cortex, including the cornu ammonis 1 (Ca1), Ca3 and dentate gyrus (DG), in the cuprizine-fed mice, and LINGO-1 antibody could partly increase the level of MBP (Figure 2D-2L).

### Reduction of proteins essential for axonal transport in the hippocampus

**Figure 3:**
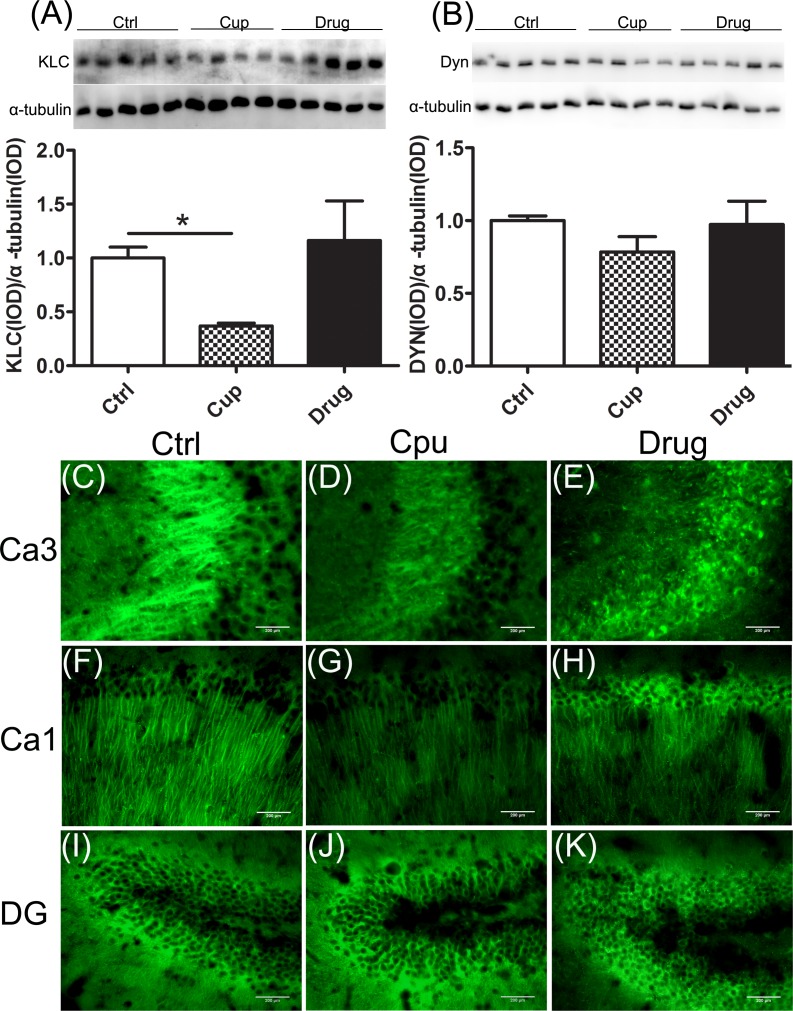
Reduction of proteins essential for axonal transport in the hippocampus **A.** KLC expression in the hippocampus was compared among the three groups. **B.** Dyn expression in the hippocampus was compared among the three groups. Figures **C.**-**K.** were displayed the immunofluorescence staining of KLC in subregions of the hippocampus, including Ca3 (C-E), Ca1 (F-H) and DG (I-K).*denotes statistical significance compared with controls (*P* < 0.05). Images were captured from stained frozen sections using a fluorescence microscope equipped with 40×objectives. Scale bar, 200μm.

Efficient axonal transport is essential to maintain neuronal function [32]. KLC is responsible for binding of cargo during fast anterograde transport, whereas dynein (Dyn) is the important proteins involve in retrograde transport [32]. We found that the level of KLC was significantly declined in the hippocampus of the cuprizine-fed mice and it was slightly increased, but not significantly, after LINGO-1 antibody treatment (Figure 3A). However, no significant change of Dyn level was measured among the three groups of mice (Figure 3B). In immunofluorescence staining, we also found decreased expression of KLC in subregions of the demyelinated hippocampal cortex, including Ca3 and Ca1, and LINGO-1 antibody could partly increase the level of KLC (Figure 3C-3H). However, the expression of KLC was similar in DG among the three groups (Figure 3I-3K).

**Figure 4:**
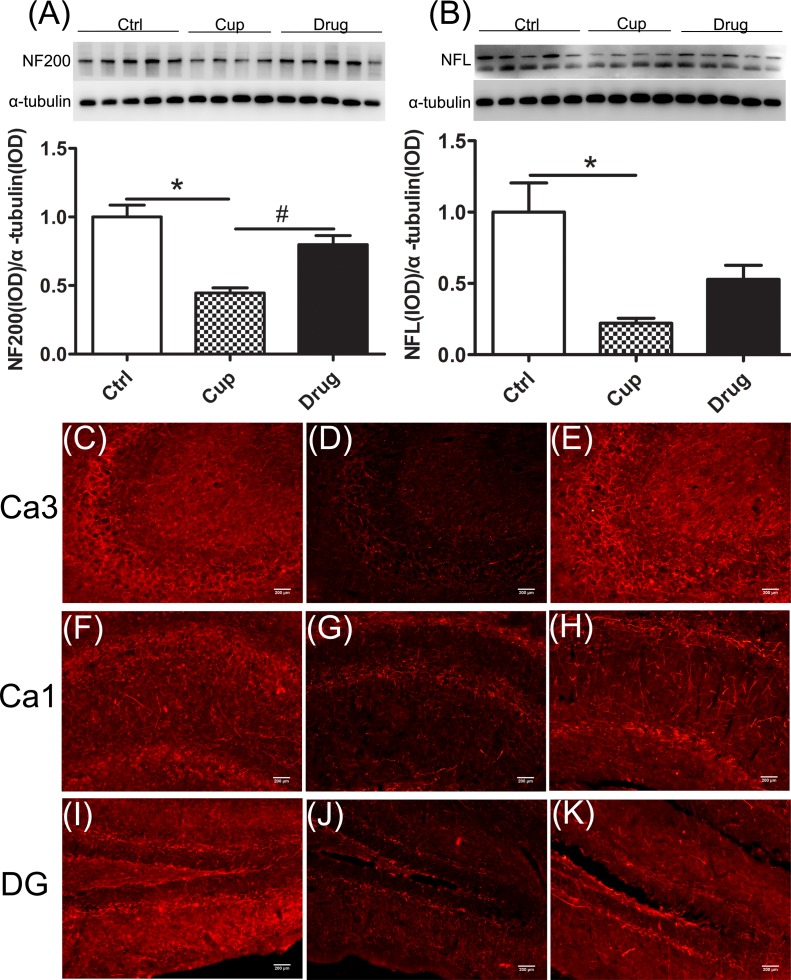
Reduction of axonal structure proteins in the hippocampus **A.** and **B.** The expression of NF200 and NFL in the hippocampus was compared among the three groups. Figures **C.**-**K.** were displayed the immunofluorescence staining of NF200 in subregions of the hippocampus, including Ca3 (C-E), Ca1 (F-H) and DG (I-K).*denotes statistical significance compared with controls (*P* < 0.05). #denotes statistical significance compared with the cuprizine-fed mice without treatment (*P* < 0.05). Images were captured from stained frozen sections using a fluorescence microscope equipped with 20×objectives. Scale bar, 200μm.

The neurofilaments (NFs) are a major component of the neuronal cytoskeleton, involved in providing structural support for the axon and regulating axon diameter, essential for the formation of axonal networks [33]. In the hippocampal cortex, the expression of NF200 in the cuprizine-fed mice was lower than that in the control mice, and after six-week LINGO-1 antibody treatment, NF200 was increased significantly (Figure 4A). Also NFL was significantly reduced in the cuprizine-fed mice and was increased but not significantly (Figure 4B) after six-week LINGO-1 antibody treatment. In immunofluorescence staining, we also found decreased expression of NF200 in subregions of the demyelinated hippocampal cortex, including Ca3, Ca1 and DG, and LINGO-1 antibody could increase the level of NF200, which was consistent with the finding of western blot (Figure 4C-4K).

### No increase of hyperphosphorylated tau and beta-amyloid in the hippocampus

**Figure 5:**
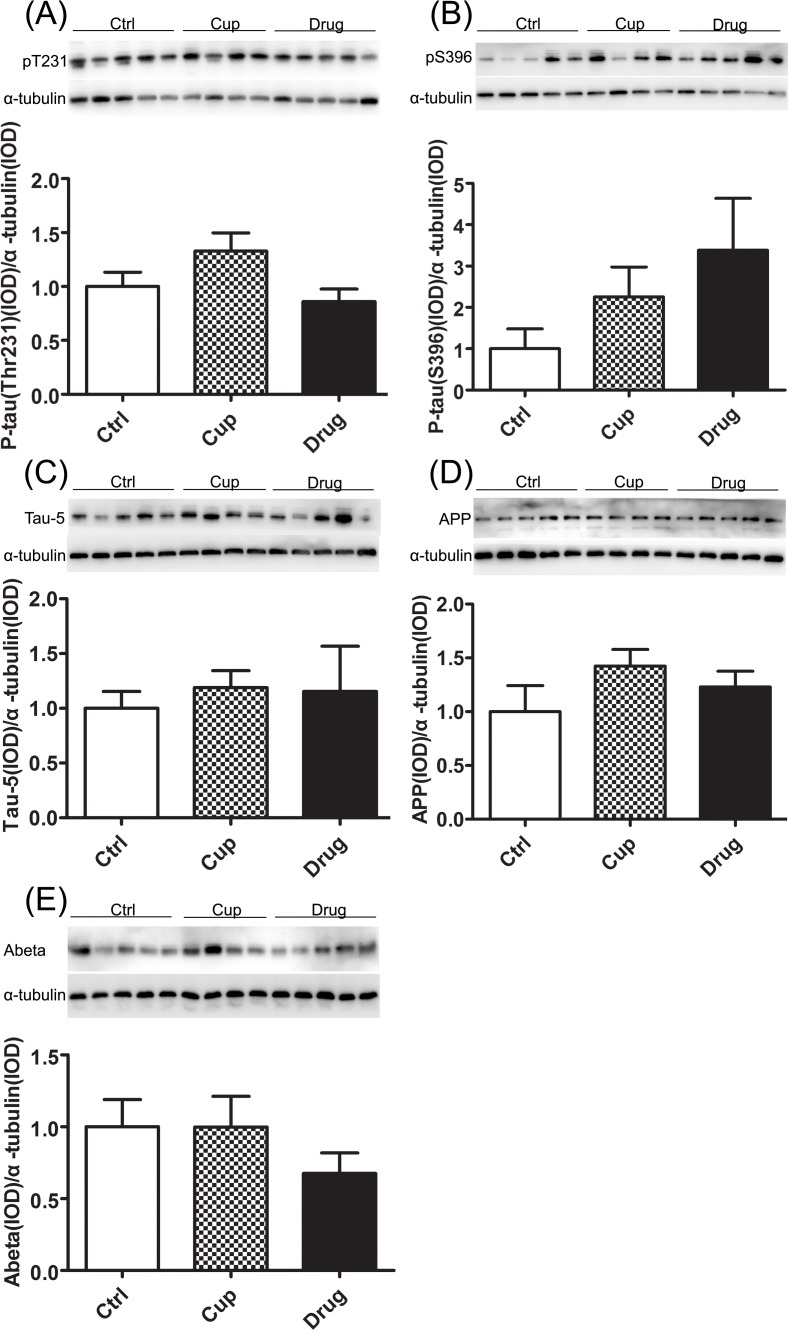
No increase of hyperphosphorylated tau and beta amyloid in the hippocampus The phosphorylated-tau antibodies PT231 **A.** and PS396 **B.** as indicated were used to measure the alteration of tau among the three groups in the hippocampus. **C.** Total level of tau, tau-5 expression was compared among the three groups. The level of APP **D.** and beta-amyloid **E.** was measured by Western-blot. *denotes statistical significance compared with controls (*P* < 0.05).

Hyperphosphorylation of tau is one of the pathological hallmarks of AD. In the research, we detected the level of phosphorylated and total tau. Tau hyperphosphorylation at the Ser396 and Thr231 epitopes was not found in the cuprizine-fed mice, displayed as the level of tau hyperphosphorylation at the Ser396 and Thr231 episodes in the cuprizine-fed mice was similar to that in the control mice, and after LINGO-1 antibody treatment, the phosphorylation of tau was also not changed (Figure 5A and 5B). Moreover, total level of tau, tau-5 was similar among the three groups (Figure 5C).

Beta-amyloid accumulation is another pathological hallmark of AD. And beta-amyloid peptide is derived from proteolytic cleavage of the amyloid protein precursor (APP) in the axon [34]. In the research, we used western blotting to detect the level of APP, and we found that there was no difference in the level of APP among the three groups (Figure 5D). And we also detected the level of beta-amyloid in the hippocampus and found that the level of beta-amyloid was similar among the three groups (Figure 5E).

### Decreased level of synaptic protein in the hippocampus

**Figure 6:**
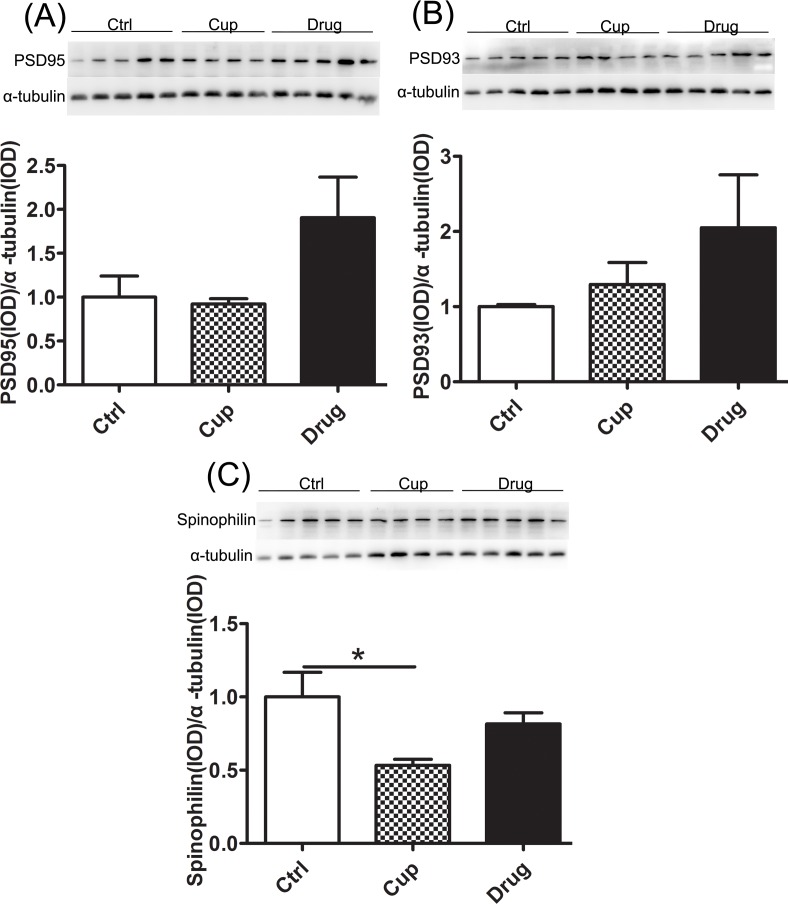
Decreased level of synaptic protein in the hippocampus **A.** and **B.** The expression of postsynaptic density proteins (PSD95 and PSD93) was compared among the three groups. **C.** The expression of Spinophilin in the hippocampus was compared among the three groups. *denotes statistical significance compared with controls (*P* < 0.05).

Postsynaptic density proteins (PSD95 and PSD93) and Spinophilin are the neural proteins essential for synaptic plasticity [35]. In the research, we found no significantly different in the expression of postsynaptic density proteins (PSD95 and PSD93) among the three groups (Figure 6A and 6B). And the expression of Spinophilin, associated to spines, was markedly decreased after ten-week cuprizine-fed and was slightly increased but not significantly in the LINGO-1 antibody treated groups (Figure 6C).

### No obvious neuronal loss in the hippocampus

**Figure 7:**
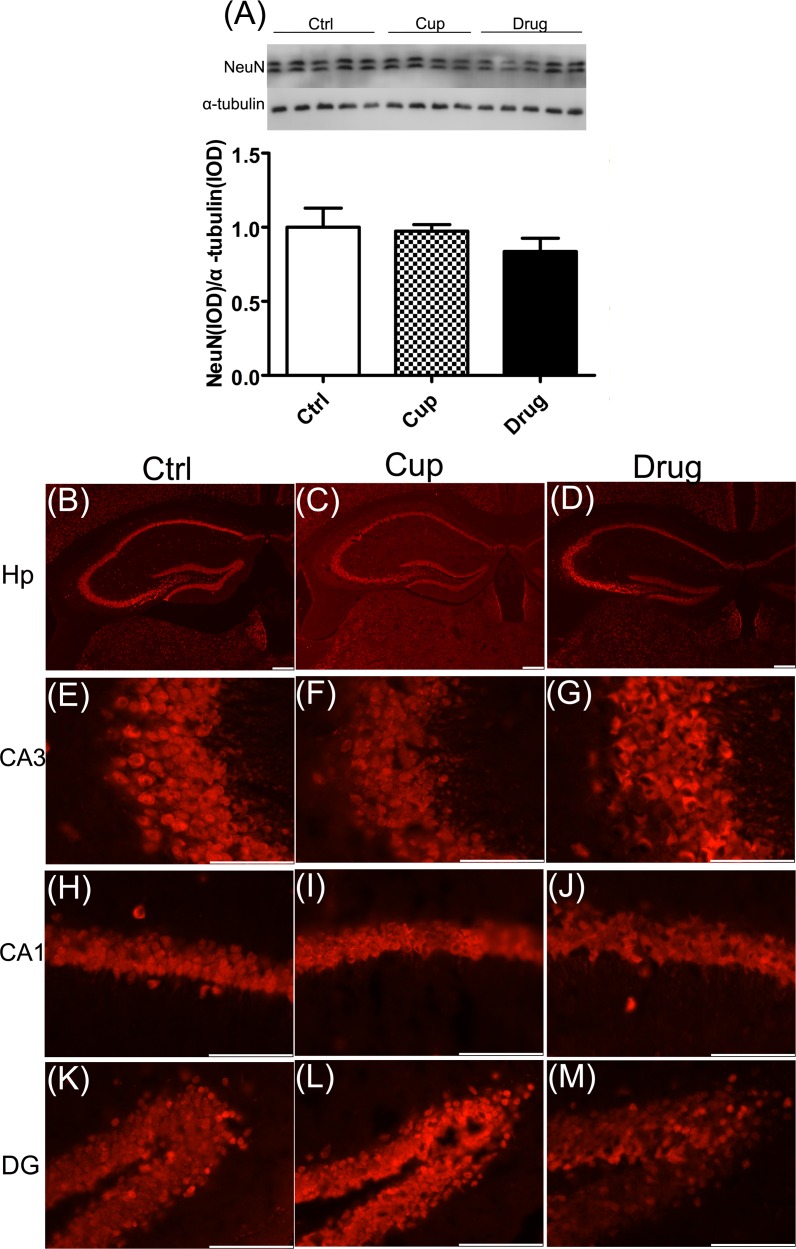
No obvious neuronal loss in the hippocampus The expression of NeuN in the hippocampus was detected by western-blot **A.** and immunofluorescence staining **B.**-**M.** respectively. Figures (B-D) were shown the expression of NeuN in whole hippocampus. Images were captured from stained frozen sections using a fluorescence microscope equipped with 4×objectives. Figures (E-M) were shown the expression of NeuN in subregions of the hippocampus, including Ca3 (E-G), Ca1 (H-J) and DG (K-M). Images were captured from stained frozen sections using a fluorescence microscope equipped with 40×objectives. *denotes statistical significance compared with controls (*P* < 0.05). Scale bar, 100μm.

Neuronal loss is also one of the main characteristics of AD. In the research, we detected the expression of NeuN, a marker of neuron, using western blot, and found no significant difference in the expression of NeuN among the three groups in the hippocampus (Figure 7A). Also, neuronal status in subregions of the hippocampus was determined by NeuN immunofluorescence staining. And the results were consistent with that of western blot (Figure 7B-7M).

## DISCUSSION

In the present study, we found the mild spatial learning impairment accompanied by the reduction of MBP in the hippocampus of the cuprizone-fed mice. After LINGO-1 antibody treatment, the learning ability of the cuprizone-fed mice was improved with an increased level of MBP. Meanwhile, the anterograde transport protein KLC, neurofilament proteins NF200 and NFL were significantly declined in the cuprizone-fed mice. After the treatment with LINGO-1 antibody, the proteins of KLC, NF200 and NFL were also increased. However, the abnormal hyperphosphorylated tau, increased beta amyloid and neuronal loss were not observed in demyelinated hippocampus of the cuprizone-fed mice. Our results suggest that demyelination might lead to the impairment of axonal transport but not cause abnormal hyperphosphorylated tau and increased beta amyloid.

Cognitive impairment is one of the common symptoms in MS patients [1]. Previous research has demonstrated that cognitive impairment is correlated to demyelination in the brain of MS patients and EAE mice [31, 36–38]. We found in our study that the cuprizone-fed mice displayed mild spatial learning deficits with the decline of MBP and the ability of learning was improved after myelin repair. Combined with previous research, our study suggests that demyelination might cause the cognitive impairment which could be reversed by promoting remyelination. MS patients also have suffered the abnormal emotion, such as depression and anxiety. Up to 60% MS patients have depression and over 30% patients have anxiety [1, 39]. However, in our research, the depression and anxiety-like behaviors were not detected in cuprizone-fed mice. It might be caused by the hyposensitivity of tests, which were used to evaluate depression and anxiety-like behaviors. Furthermore, to our acknowledgement, the variance within every group is fairly high in our research, and this may mean that we underestimate the significance of differences among groups.

Myelin wraps around neuronal axons and it is essential to functional integrity and long-term survival of the neuronal axons [40, 41]. The impairment of myelin can cause different type of axonal pathology. Previous research has demonstrated that there is a tight relationship between the myelin and axonal transport [12, 42]. Kinesins are known as the main molecular motors that drive cargoes from the neuronal cell bodies to the distal nerve terminals along the microtubule [43]. KLC is one of components of kinesin-I and may involve in coupling cargo to the heavy chain or modulating its ATPase activity [43, 44]. Previous study has suggested that demyelination may lead to the impairment of axonal transport in the MS [12]. We previously find that there is a decreased level of KLC in the demyelinated parahippocampal cortex (PHC) in the EAE mice, and it can be restored after remyelination treatment [31]. In the present study, we also found the reduction of KLC in demyelinated hippocampus of cuprizone-fed mice, which could be partly restored by LINGO-1 antibody.

NFs are major components of the neuronal cytoskeleton, which are the basis for specialized axonal structures and required for axonal transport [33, 43, 45, 46]. Previous study has demonstrated that the neurofilament content is increased in the myelinated axonal segment, compared with the unmyelinated part [47]. The reduction of axonal NFs is observed in demyelinated PHC of EAE mice [31]. Furthermore, the impaired NFs can be recovered accompany with the repair of myelin [31]. In the present study, we found the decline of NF200 and NFL in demyelinated hippocampus of cuprizone-fed mice. And also increased level of NF200 and NFL was observed in accordance with myelin repair.

Axonal transport associated proteins and normal axonal structure are crucial to the axonal transport in neurons [43]. In our research, we observed that both axonal transport associated protein (KLC) and axonal structural proteins (NF200 and NFL) were decreased in demyelinated hippocampus of cuprizone-fed mice and were partly increased after myelin repair. Furthermore, we did not find neuronal loss in demyelinated hippocampus of the cuprizine-fed mice, which suggests that the decline of KLC, NF200 and NFL was not simply due to the loss of neurons in the hippocampus. Our results suggest that demyelination might lead to the impairment of axonal transport in the hippocampus, which could be ameliorated by remyelination.

The hyperphosphorylation of tau and beta-amyloid accumulation are important hallmarks of AD. Much research, including clinical and preclinical research, has detected the level of hyperphosphorylated tau and beta amyloid in MS patients and animal models. However, the results are inconsistent [48–51]. In our research, we neither found the hyperphosphorylation of tau at pS396 and pT231, nor observed the increased level of beta amyloid in demyelinated hippocampus of the cuprizone-fed mice. Based on our finding and the previous research, we infer that demyelination alone can not induce AD-like pathology, such as hyperphosphorylated tau and increased beta-amyloid, in a short time.

In conclusion, our results suggested demyelination might cause dysfunction of axonal transport. However, the impairment of myelin alone did not lead to hyperphosphorylation of tau, increase of beta amyloid and neuronal loss, which are important hallmarks of AD.

## MATERIALS AND METHODS

### Animals

Adult (C57BL/6, eight-week-old, male) mice were purchased form the animal research center of shanghai laboratory and were housed at 22°C-24°C. Food and water were available ad libitum. Animals were cared for in accordance with the National Institutes of Health Guidelines for Animal Care. All experimental procedures were approved by Animal Care and Use Committee in Southeast University.

At the beginning of the research, the mice were randomly divided into three groups. Three groups included the control group (*n* = 17), the non-pharmacologic treatment Cuprizine-fed group (*n* = 17) and the pharmacologic treatment Cuprizine-fed group (*n* = 17). 8-Week-old C57/Bl6 mice were fed with 0.2% (w/w) cuprizone (bis-cyclohexanone oxaldihydrazone) (Sigma) in ground breeder chow for about ten weeks.

### LINGO-1 antibody treatment

The LINGO-1 antibody in the research was generated based on the method of Mi et al [29], but using the BALB/c strain of mice. In our previous research, we have demonstrated that the LINGO-1 antibody is specifically binding to the LINGO-1 protein [31]. LINGO-1 antibody treatment was begun in the third week, for significant demyelination is detected in the third week in the Cuprizine-fed mice [26]. For systemic drug delivery, the mice in the treatment group received intraperitoneal injections of 10 mg/kg LINGO-1 antibody once every six days like our previous research [31]. The mice, in the other two groups, were administered 0.9% NaCl once every six days. During LINGO-1 antibody treatment, the cuprizine was continued to feed the mice and it did not stop until the mice were killed.

### Behavioral analyses

At weeks 9 to 9.5, we tested the behaviors of mice. Before the behavioral tests, the mice were taken to the new environment to acclimate for two days. The order of the behavioral tests was from low-stimulation experiments to high-stimulation, as follows: the elevated plus maze, open field test, sucrose preference test (low-stimulation experiments), and Morris water maze (high-stimulation one).

### The elevated plus maze (EPM)

The elevated plus maze (EPM) is an experiment, which is widely used to assess anxiety in rodents. The EPM test was conducted following the previously described way [31]. Briefly, mice were placed in the center of the maze facing an open arm and allowed to freely explore the EPM for five minutes. The percentage (%) of open arm distances and the percentage (%) of time spent on the open arms were recorded to measure the anxiety of mice. Decreased open arm activities indicate increased anxiety levels in the EPM. Between each trial, the maze was wiped clean with a damp sponge and dried with paper towels.

### Open field test

The open field test is used to assess the general locomotor activity and anxiety of rodents. The test was conducted following the previously described way [31]. Each mouse was placed in the center of the open field apparatus (50 cm × 50 cm × 60 cm) and can move freely for 5 min. The average speed and time/distance in the center was recorded to measure the locomotor activity and anxiety levels. Between each trial, the maze was wiped clean with a damp sponge and dried with paper towels.

### Sucrose preference test

The sucrose preference test is a way, used to test the level of anhedonia in mice. The test was conducted following the previously described way [31]. The mice were habituated to 2% sucrose solution for one day prior to the start of the experiment. On the test day, the mice were housed singly with ad libitum food and two bottles—one with water and the other with 2% sucrose solution—for 24 hours. The bottles were reversed halfway through the time to avoid a side preference. The weights of the two bottles were recorded to calculate the sucrose consumption. The preference for the sucrose solution was calculated as a percentage of total liquid consumed. The sucrose preference rate was calculated using the following formula: sucrose preference rate = sucrose consumption / (water consumption + sucrose consumption) × 100%.

### Morris water maze

The water maze test was a good way to test spatial learning and memory ability [52]. The maze consisted of a 1.2-m diameter circular pool filled with water (22°C) that was made opaque by the addition of non-toxic, water-based white food coloring. A circular Plexiglas escape platform (10 cm in diameter) was located in the center of one of the quadrants of the pool. The experiment consisted of two phases including five consecutive training days and one detecting day. The animals underwent four trials over the training days with the platform submerged 1.5 cm below the surface of the water (60s maximum trial duration; 20-30 min interval). The latency to reach the platform was analyzed to assess learning in the mice. On the last day, mice were tested with a single trial without the platform, starting from the opposite quadrant of the platform for 60 s. The percentage of the distance and time in the platform quadrant was measured to evaluate the memory performance.

### Western blot

Mice were killed after the behavioral tests. Mice from each group were deeply anesthetized with chloral hydrate and perfused transcardially with ice-cold 0.9% saline. The brains were dissected from the skulls, and the dorsal hippocampus was dissected under an anatomical microscope based on the stereotaxic coordinate.

Tissues were processed for homogenization and sonication on ice in lysis buffer containing 50 mM Tris-base (pH7.4), 150 mM NaCl, 1% Triton X-100, 1% sodium deoxycholate, 0.1% SDS, sodium orthovanadate, sodium fluoride, EDTA, leupeptin, 0.5 mM sodium vanadate, 1% NP-40, 0.1 mM phenylmethylsulfonyl fluoride, 1 μg/mL aprotinin, and 1 μg/mL leupeptin supplemented with protease inhibitors. The extracts were then centrifuged at 13,000 × g for 20 minutes at 4°C. The total amount of protein in the supernatant was determined with the bicinchoninic acid method using the Bio-Rad Protein Assay kit (Bio-Rad Laboratories, Hercules, CA, USA) according to the manufacturer's instructions. Next, the supernatant was diluted 5:1 with sample buffer (6× concentrate; Beyotime, China), then the samples were heated for 5 minutes at 100°C and stored at −20°C. Equal amounts of protein (5 μg per lane) were resolved on 8%, 10%, 12% and 15% SDS-polyacrylamide gels. Following the electrophoresis, the proteins were transferred to Immobilon-P Transfer membranes (Millipore, Billerica, MA, USA). The membranes were probed overnight at 4°C with the following antibodies and dilutions: anti-MBP (anti-rat monoclonal; 1:2000; Abcam), anti-CNPase (anti- rabbit polyclonal; 1:500; bioworld), anti-PLP(anti-mouse monoclonal; 1:500; Abcam), anti-dynein (anti-mouse monoclonal; 1:800; EMD millipore), anti-KLC (anti-rabbit polyclonal; 1:500; Santa), anti-NF200 (anti-rabbit polyclonal; 1:2000; Sigma), anti-NFL (anti-goat polyclonal; 1:400; Santa), anti-PSD95(anti-rabbit polyclonal; 1:1000; Abcam), anti-PSD93(anti-rabbit polyclonal; 1:1000; Abcam), anti-Spinophilin/Neurabin2(anti-rabbit polyclonal; 1:1000; Abcam), anti-βAPP(anti-rabbit polyclonal; 1:500; bioworld), anti-Aβ(anti-mouse monoclonal; 1:500; Covance), anti-tau-5 (anti-mouse polyclonal; 1:1000; Abcam), anti-P-tau(S396) (anti-rabbit polyclonal; 1:1000; Abcam), anti-P-tau(thr231) (anti-rabbit polyclonal; 1:1000; Abcam), anti-NeuN (anti-rabbit polyclone; 1:200; EMD millipore) and anti-α-tubulin (anti-rabbit monoclonal; 1:2000; KeyGEN Biotech). After rinsing, the membranes were incubated with the secondary antibody conjugated with horseradish peroxidase (HRP) at dilutions of 1:5000 for 60 minutes at room temperature. Signals were detected using an enhanced chemiluminescence kit (ECL, Millipore, Billerica, MA, USA). The signal strength was quantified using a gel pro plus imaging analyzer. The average background density was subtracted, and the integral optical density values (IOD) were measured.

### Immunofluorescence

Mice were killed after the behavioral tests. Mice from each group were deeply anesthetized with chloral hydrate and perfused transcardially with ice-cold 0.9% saline, followed by 4% paraformaldehyde. The brains were dissected from the skulls, post-fixed with 4% paraformaldehyde over night, followed by 10%, 20% and 30% sucrose solutions, each for at least 16 hours. Brain tissue was embedded in Tissue Freezing Medium (Leica, Germany), frozen at −80°C and cut with a Leica microtome into 20-μm coronal sections. Frozen sections were used to observe the expression of MBP, KLC and NF200. Neuronal status in the hippocampus was determined by NeuN immunofluorescence. Sections were incubated over night at 4°C with primary antibodies: anti-MBP (anti-rat monoclonal; 1:200; Abcam), anti-KLC (anti-rabbit monoclonal; 1:50; Abcam), anti-NF200 (anti-rabbit polyclonal; 1:200; Sigma), anti-NeuN (anti-rabbit polyclone; 1:200; EMD millipore). Following the incubation with primary antibodies, sections were washed and incubated for 2 h at room temperature with secondary antibody: donkey Alexa Fluor 488 F(ab) anti-rat IgG, goat Alexa Fluor 488 F(ab) anti-rabbit IgG, or goat Alexa Fluor 592 F(ab) anti-rabbit IgG. Images were captured from stained frozen sections using a fluorescence microscope.

### Statistical analysis

The data were presented as the means ± SEM. A one-way ANOVA with Tukey's post hoc test or least significance difference (LSD) test was used to determine statistical significance. *P* < 0.05 was set as the cutoff for statistical significance. The statistical analyses were performed using GraphPad Prism 4 software and SPSS 18.
